# A Modified Coupled Spectrophotometric Method to Detect 2-5 Oligoadenylate Synthetase Activity in Prostate Cell Lines

**DOI:** 10.1186/s12575-016-0038-x

**Published:** 2016-03-17

**Authors:** Sushma M. Bhosle, Aisha Hunt, Jaideep Chaudhary

**Affiliations:** Department of Biological Sciences, Centre for Cancer Research and Therapeutic Development, Clark Atlanta University, Atlanta, GA 30314 USA

**Keywords:** 2-5 oligoadenylate synthetase, Pyrophosphate assay, Prostate cancer

## Abstract

**Background:**

2’-5’ oligoadenylate synthetases (OAS) are interferon inducible enzymes that polymerizes ATP to 2’-5’-linked oligomers of adenylate (2-5As). As part of the innate immune response, these enzymes are activated by viral double stranded RNA or mRNAs with significant double stranded structure. The 2-5As in turn activate RNaseL that degrade single stranded RNAs. Three distinct forms of OAS exist in human cells (OAS1, 2 and 3) with each form having multiple spliced variants. The OAS enzymes and their spliced variants have different enzyme activities. OAS enzymes also play a significant role in regulating multiple cellular processes such as proliferation and apoptosis. Moreover, Single nucleotide polymorphisms that alter OAS activity are also associated with viral infection, diabetes and cancer. Thus detection of OAS enzyme activity with a simple spectrophotometric method in cells will be important in clinical research.

**Results:**

Here we propose a modified coupled spectrophotometric assay to detect 2-5 oligoadenylate synthetase (OAS) enzyme activity in prostate cell lines as a model system. The OAS enzyme from prostate cancer cell lysates was purified using Polyinosinic: polycytidylic acid (poly I:C) bound activated sepharose beads. The activated OAS enzyme eluted from Sepharose beads showed expression of p46 isoform of OAS1, generally considered the most abundant OAS isoform in elutes from DU14 cell line but not in other prostate cell line. In this assay the phosphates generated by the OAS enzymatic reaction is coupled with conversion of the substrate 2-amino-6-mercapto-7-methylpurine ribonucleoside (methylthioguanosine, a guanosine analogue; MESG) to a purine base product, 2-amino-6-mercapto-7-methylpurine and ribose1-phosphate via a catalyst purine nucleoside phosphorylase (phosphorylase) using a commercially available pyrophosphate kit. The absorbance of the purine base product is measured at 360 nm. The higher levels of phosphates detected in DU145 cell line indicates more activity of OAS in this prostate cancer cell line.

**Conclusion:**

The modified simple method detected OAS enzyme activity with sensitivity and specificity, which could help in detection of OAS enzymes avoiding the laborious and radioactive methods.

## Background

The interferon inducible anti-viral 2-5 oligoadenylate synthetase (OAS) enzyme is part of the cellular innate immune response [1–3]. There are eight to ten OAS isoforms due to alternative splicing and gene duplications [4, 5]. In humans, the OAS gene family is composed of four genes on locus 12q24.1-q24.2: hOAS1, hOAS2, hOAS3 and hOASL [6, 7]. A pathogen-associated molecular pattern (PAMPs) such as dsRNA or mRNA with significant double stranded structures stimulates OAS to catalyze the polymerization of ATP to form 2’-5’-linked oligomers of adenylate (2-5As) [4, 8]. The subsequent elongation reaction is (n + 1) ATP → Ppp5’A(2’p5’A)n + nPPi [9]. 2-5A functions through its receptor, the 2-5A-dependent ribonuclease (RNaseL), a ubiquitous 83-kDa protein that dimerizes into its catalytically active form upon binding 2-5A [4, 10, 11]. The activated RNaseL cleaves the single stranded RNA with UU or UA dinucleotide at the 3’ end [4, 8, 12, 13]. Thus activated OAS-RNaseL system degrades viral and cellular RNA and subsequently inhibits protein synthesis [14]. Earlier studies also showed that OAS-RNase L system triggers apoptosis via mitochondrial pathway [15, 16]. It is also known that OAS silencing attenuates interferon induced anti-proliferative and antiviral signaling and stimulates anchorage independent cell growth [8]. The exogenous recombinant porcine OAS1 exhibits broad-spectrum antiviral activity through the activation of the IFN system that is independent of RNaseL [17]. Thus OAS1 dependent polymerization of ATP regulates multiple cellular functions that are dependent or independent of RNaseL.

OAS1 gene single nucleotide polymorphism (SNP) is associated with wide variety of health related disorders such as hepatitis C infection [18, 19], West Nile virus infection [20], SARS [21] and Type1 diabetes [22]. The haplotype variations in OAS1 gene also confer susceptibility to multiple sclerosis [23]. A recent study also showed a non-synonymous SNP rs2660 (G-A) is associated with racial disparities in prostate cancer [24]. It is being demonstrated that OAS1 gene alternative splicing is in part due to SNP at the splice acceptor site (rs10774671) generates p46 and p48 isoforms with different ATP polymerization activity [25]. Collectively, SNPs in OAS gene results in variation in activity of the enzyme which could be associated increased susceptibility for several disorders listed above. Thus, development of a simple and accurate assay to detect the activity of OAS1 enzyme is of significant clinical interest.

Earlier, OAS activity was analyzed by determining the inhibitory effect of 2-5A on protein translation [26], detection of 2-5A polymers in the presence of radioactive ATP [27–29] and/or cleavage of radioactive 2-5A with alkaline phosphatase to cleave the triphosphate group at the end of the core nucleotide. Later, Justesen et al. developed a method to separate radioactive ATP and 2-5A by PEI-cellulose thin layer chromatography without the use of alkaline phosphatase [30]. The method was subsequently improved to measure inorganic pyrophosphate (PPi) through a spectrophotometric assay [9]. This method is a coupled enzymatic reaction utilizing three enzymes UDP-Glc pyrophosphorylase, phosphoglucomutase and glucose-6-phosphate dehydrogenase. The coupled reaction results in formation of NADPH which is measured by fluorescence spectroscopy [9]. The use of extensive OAS1 chromatographic purification steps, expensive reagents, and series of reactions utilized in this methodology to detect OAS enzyme has made it more cumbersome, laborious and expensive.

The detection of inorganic phosphate released from biological systems, such as GTPases and ATPases has been developed earlier [31]. The phosphate generated during the enzymatic reaction converts the substrate 2-amino-6-mercapto-7-methylpurine ribonucleoside (methylthioguanosine, a guanosine analogue; MESG) to a purine base product, 2-amino-6-mercapto-7-methylpurine and ribose1-phosphate via a catalyst purine nucleoside phosphorylase. This conversion causes a shift in absorbance from 330 nm of substrate (MESG) to 360 nm of purine base product (2-amino-6mercapto-7-methylpurine) [31]. Further this method was modified to detect pyrophosphates generated in several biochemical reactions by converting the pyrophosphates to inorganic phosphates by a phosphatase. Thus the reaction producing pyrophosphate can be coupled to inorganic phosphate detection [31]. This modified method of detecting pyrophosphate has been known to measure activity of acetyl CoA synthetase and amino-acyl-tRNA synthetase activity which are also involved in pyrophosphate generation [32, 33]. The modified pyrophosphate detection method is also employed in hydroxamate-MESG assay which measures the activity of adenylating enzyme via measurement of pyrophosphate generated from ATP during the activation of carboxylic acid to form acyl-adenylate intermediate. The coupled hydroxamate assay characterizes the adenylate enzyme that do not undergo rapid ATP –PPi exchange during the acylation of the protein substrate like hydroxamate [34].

In this study, the OAS enzyme from prostate cell lysates was first affinity purified and activated with the use of sepharose beads conjugated with Poly I:C (Polyinosinic: polycytidylic acid). The purified enzyme was eluted with elution buffer resulting in the generation of inorganic pyrophosphate. We exploited the polymerization reaction which converts ATP in the elution buffer to 2-5A and pyrophosphates by poly I:C affinity purified OAS and coupled the OAS enzyme reaction generating pyrophosphate with the modified method of detecting pyrophosphates based on conversion of MESG substrate to purine based product 2-amino-6-mercapto-7-methylpurine by phosphates. This method for detecting OAS enzyme is less laborious, nonhazardous and cost effective over the other known cumbersome and multistep methods of detecting OAS.

## Methods

### Cell Lines

All the cell lines were purchased from ATCC. The normal prostate epithelial cell line RWPE1 (ATCC) was cultured in K-SFM (keratinocyte serum free medium) supplemented with BPE (Bovine pituitary extract) and EGF (Epidermal growth factor) as per the supplier’s recommendation. The 293T were cultured in high glucose DMEM media. The prostate cancer cell lines LNCaP were culture in RPMI, DU145 in F12 medium. The cells were incubated at 37 °C and 5 % CO2 under sterile conditions.

### Reagents

Sodium periodate (NaIO4), Sodium azide, Sepharose and Cyanoborohydride coupling buffer were obtained from Sigma-Aldrich. Poly I:C and EnzChek pyrophosphate Kit were purchased from Invitrogen, Cell lysis buffer M-PER were obtained from Pierce.

### Affinity Purification for OAS Enzyme

Sepharose beads (Sigma) were activated by 10 mM sodium periodate solution in 1 M sodium acetate (pH 5.5). One bed volume of sepharose beads were mixed with activation solution at room temperature for 30 min followed by washing twice with distilled water.

### Poly I:C Coupling to Activated Sepharose Beads

**Fig. 1:**
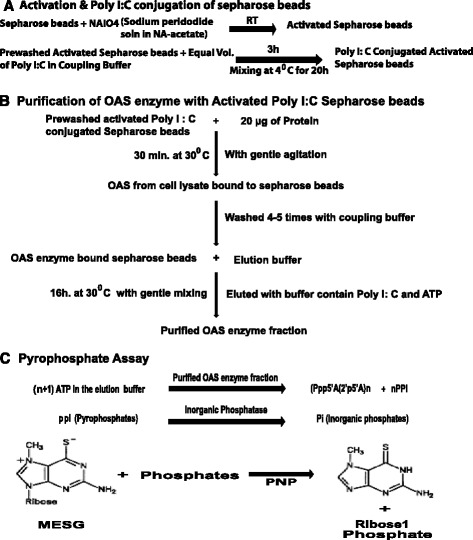
Method to measure OAS enzyme activity. Schematics of **a** Activation and Conjugation of Poly I:C to sepharose beads, **b** Purification of OAS enzyme and **c** OAS enzymatic reaction coupled to pyrophosphate reaction

The poly I:C coupling buffer was prepared by dissolving 2 mg of Poly I:C in 1 ml of cyanoborohydride coupling buffer. One ml bed volume of periodate activated sepharose beads were washed twice and mixed with equal volume of poly I:C coupling buffer at room temperature (RT) for 3 h, followed by gentle mixing at 4 °C for 20 h. The beads were then centrifuged at 500 X g for 2 min and washed with 4 ml of coupling buffer. After washing, the residual reactive groups were blocked by incubating the beads with coupling buffer containing 0.5 M Tris-HCl (pH 7.4) for 60 min at RT with gentle agitation. Finally, the beads were washed three times with 10 ml PBS and then stored at 4 °C in PBS (pH-7.4) containing 0.02 % sodium azide until further use (Fig. 1a).

### Purification of OAS Enzyme with Poly I:C Sepharose Beads

Cells were lysed with cell lysis buffer M-PER. Total cellular protein was quantified with Bradford assay (Bio-Rad). Proteins (20 μg) diluted with dH_2_O in total volume of 20 μl were mixed with equal volume of Poly I:C conjugated Sepharose beads (Fig. 1b). Poly I:C bound Sepharose beads stored in sodium azide were prewashed with PBS before use. The cell lysate and Sepharose beads mixture was incubated for 30 min at 30 °C with gentle agitation for adsorption and activation of OAS enzyme on Poly I:C conjugated and activated Sepharose beads. Following incubation, the beads were further washed several times with PBS. Finally, OAS enzyme bound to poly I:C was eluted by incubating Sepharose beads with 25 μl of elution buffer [20 mM of magnesium acetate, 1.5 mM of ATP, 1 mM DTT, 25 mM HEPES (pH 7.5), 50 μg/ml of poly I:C] for 16 h at 30 °C with gentle mixing. The beads were then centrifuged at 500 x g for 2 min and the supernatant containing the elution buffer and the active enzyme product was separated and store at −20 °C. The elution buffer contains Poly I:C which can elute OAS from Sepharose beads by competitive affinity binding.

### Pyrophosphate Assay

Pyrophosphate (PPi) generated in a polymerization reaction of ATP by OAS was detected using an EnzChek pyrophosphate kit. Assays were performed as per the manufacturer’s instructions with some modifications. Briefly all the assay components: 20x reaction mixture, MESG substrate, OAS enzyme/inorganic phosphate for standard curve, purine nucleoside phosphorylase (PNP), and inorganic pyrophosphatase were mixed in a total reaction volume of 100 μl. The reaction mixture was incubated at room temperature for 15–20 min. The absorbance of MESG was first measured at 330 nm. After addition of the enzyme, the conversion of MESG to the product 2-amino-6-mercapto-7-methylpurine by phosphate (Pi) was measured at 360 nm. A standard curve with increasing concentration of inorganic phosphates was expressed in terms of increasing absorbance of the product at 360 nm. A wavelength scan for shifts in absorbance as the reaction proceeded from approximately 330 nm to 360 nm in the presence of Sepharose conjugated Poly I:C alone was measured as a baseline.

### Western Blotting

The purified cell lysates obtained by Poly I:C conjugated activated Sepharose beads was investigated for the expression of p46 isoform of OAS1. Briefly, 20 μg of purified and crude lysates from 293T, DU145, LNCaP and RWPE1 were loaded on 10 % SDS-PAGE gel and transferred on nitrocellulose paper. The p46 OAS1 isoform was detected by goat primary antibody against p46 (OAS1 (A-17), goat polyclonal, Santa Cruz Biotechnology) using standard western blot protocol. The blots were analyzed using Fujifilm image analyzer (LUMINESCENT IMAGE ANALYZER LAS-300). GAPDH was used as a loading control. DU145 cell lysates purified using inactive Sepharose beads with no Poly I:C conjugated were used as a negative control. The Poly I:C unbound fraction of cell lysates obtained were also tested for detecting the fraction of P46 active isoform of OAS.

### Statistical Analysis

The Statistical significance was calculated by one tailed unpaired *t*-test. The OAS activity in cell lines was compared to the activity in lysates from control DU145 cells which was purified by un-activated Sepharose beads without Poly I:C. The assay was performed thrice in triplicates. The P value is represented as *** for <0.005 and as * for value < 0.05 is shown.

## Result and Discussion

Interferon inducible and dsRNA activated 2-5 OAS is a critical component in the enzymatic reaction to form 2-5A polymer which is an activator of RNaseL. Together, the 2-5A/RNaseL system forms a vital part of innate immune system [4, 10, 11]. The activated RNaseL degrades the viral RNA and provides defense against infected viruses [14]. The variation in enzyme activity is attributed to non-synonymous single nucleotide polymorphism at the splice acceptor site in the last exon for at least OAS1. This variant of OAS1 enzyme is associated with increased viral infection due to lack of OAS enzyme function [25].

Here, we developed a simple method of detecting OAS by coupling pyrophosphates generated during the polymerization of ATP to form 2-5A in presence of the enzyme OAS to the conversion of MESG substrate to purine based product with absorbance peak at 360 nm by using a commercially available kit. The enzyme in the cell lysates was activated and purified using NaIO4 activated and poly I:C conjugated sepharose beads (Fig. 1). The purified enzyme polymerizes ATP and releases pyrophosphates. Further, pyrophosphates are converted to phosphate in the presence of inorganic phosphatase to the reaction. In the presence of ribonucleoside phosphorylase (PNP), phosphates react with substrate MESG and converts it to 2-amino-6-mercapto-7-methylpurine and ribose 1- phosphate (Fig. 1). Earlier, this method has been utilized to measure phosphates released by enzymes like actin activated myosin ATPase [31] and phosphorylase kinase [35] and AcsD, an acyladenylate-forming enzyme from Pectobacterium chrysanthemi [36]. This method has advantage over several earlier less sensitive assay involving multistep reactions [37].

### Detection of OAS1 p46 Isoform in Poly I:C Affinity Beads Purified Lysates

**Fig. 2:**
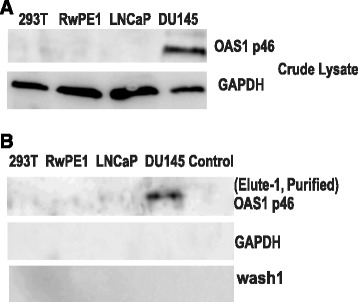
OAS1 protein expression in crude and purified lysates of prostate cancer cell line. OAS1 p46 isoform expression in lysates from 293T, RwPE1, DU145 and LNCaP cell line. **a** OAS 1 expression in 20 μg crude lysates (*upper panel*) and GAPDH (*lower panel*) was used as a loading control. **b** OAS 1 expression in lysates purified from 20 μg of crude lysates with activated poly I:C bound sepharose beads (*upper panel*), GAPDH (*middle panel*) was used as a loading control and elute-1 collected during washing to remove the unbound OAS enzyme and OAS free cell lysates. DU145 lysates purified with unactivated sepharose beads with no poly I:C bound to it was used as a negative control

As described earlier, we purified OAS from the cell lysates using affinity capture technique (see schematic in Fig. 1). The Poly I:C conjugated and activated sepharose beads to purify OAS enzymes from cell lysates is well established and described earlier [38]. The bound enzyme was eluted and was analyzed for the expression of p46 isoform of OAS1 before testing the purified lysates to detect OAS enzyme activity. The OAS1 (p46) isoform protein expression was detected in crude cell lysates from DU145 cell lines but was absent or non-detectable in 293T, LNCaP and RWPE1 cell lines. The 293T cell line was used as a negative control as it lacks the active form of OAS1 (Fig. 2a). Figure 2b shows the OAS1 (p46) isoform expression in the cell lysates purified by Poly I:C conjugated Sepharose beads. OAS1 (p46) was detected in the poly I:C sepharose affinity purified lysates from DU145 cells but not in the purified lysates from other prostate cell lines and 293T cells. Thus the presence of OAS1 in purified lysates of DU145 suggested that the poly I:C conjugated sepharose affinity purification system is functional and results in the enrichment of OAS enzyme. The absence of detectable OAS enzyme from the DU145 cell lysate purified with inactivated Sepharose beads with no bound poly I:C used as a negative control further supports the specificity of the affinity purification system. Furthermore, no GAPDH expression was observed in the purified fraction suggesting the poly I:C conjugated sepharose beads specifically captured only OAS (Fig. 2b). The first wash flow through, collected after binding of OAS enzyme to poly I:C-sepharose beads resulted in undetectable p46 OAS1 enzyme suggesting that the affinity column captured essentially all the enzyme present in the cell lysate (Fig. 2b). These purified fractions were used for detecting OAS enzyme assay by simple and rapid pyrophosphate assay.

### Measurement of Pyrophosphate

**Fig. 3:**
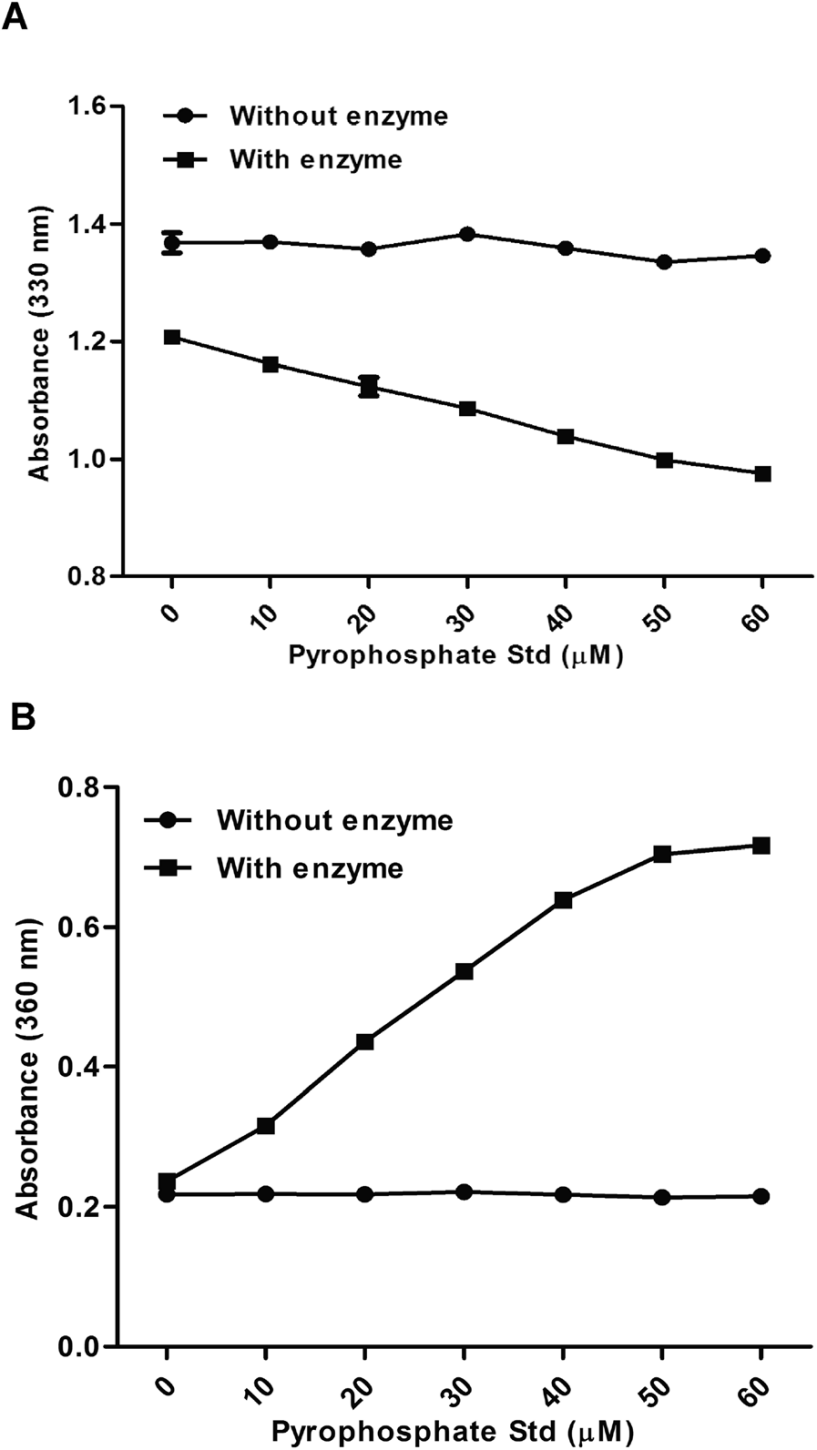
Standard curve for measurements of pyrophosphates. **a** Absorbance of MESG substrate at 330 nm with increasing concentration of pyrophosphates (10–60 μM) with or without PNP and inorganic phosphatase. **b** Absorbance of purine based product, 2-amino-6-mercapto-7-methylpurine at 360 nm with increasing concentration of pyrophosphates (10–60 μM) with or without PNP and inorganic phosphatase

The pyrophosphate assay uses MESG substrate which reacts with phosphate in presence of enzyme purine ribonucleoside phosphorylase (PNP). The resulting product has an absorbance at 360 nm. A standard curve was constructed to detect the change in absorbance with increasing concentration of pyrophosphate. A gradual decrease in MESG absorbance with increasing concentration of pyrophosphate at 330 nm was observed after addition of enzyme PNP and inorganic phosphatase (Fig. 3a). Correspondingly, with the increasing concentration of the pyrophosphate, an increase in the absorbance of the product 2-amino-6-mercapto-7-methylpurine was observed at 360 nm (Fig. 3b). The activity of OAS in the Poly I:C conjugated affinity beads purified cell lysate in terms of the pyrophosphate generated during enzymatic reaction can be detected by measuring increase in absorbance of the product or decrease in absorbance of the substrate. The amount of pyrophosphate generated can then be determined from the standard curve (Fig. 3a and b).

### Measurement of OAS Enzyme Activity in Purified Prostate Cell Lysates

**Fig. 4:**
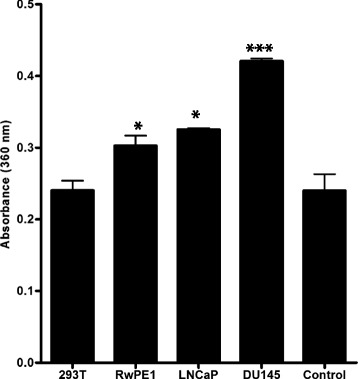
Enzyme activity in prostate cancer cell line. OAS enzyme assay measurement with pyrophosphate assay in lysates purified from 293T, RwPE1, LNCaP, DU145 and control which is lysate from DU145 cell purified with unactivated sepharose beads with no poly I:C. Shift in absorbance at 360 nm after completion of reaction post addition of PNP and inorganic phosphatase. Assay was done thrice in triplicates. Statistical significance is shown as *** for *p* < 0.005 and * for *p* < 0.05

The purified lysates tested for expression of p46 isoform of OAS1 (Fig. 2) were analyzed for OAS enzyme activity. The enzyme activity measured in purified prostate cell lysates and the negative control 293T cells which lack endogenous OAS1 by pyrophosphate assay kit. The enzymatic conversion of 2-amino-6-mercapto-7-methylpurine ribonucleoside (MESG) substrate to 2-amino-6-mercapto-7-methylpurine is directly proportional to the amount of inorganic phosphate produced by the OAS enzyme. There is a significant increase in absorbance of product 2-amino-6-mercapto-7-methylpurine at 360 nm in DU145 cell lines showing high OAS enzyme activity in this cell lines as compared to the other two prostate cell lines which did not show any enzyme activity (Fig. 4). OAS enzyme activity was not observed in negative control 293T purified cell lysate and the DU145 cell lysate passed through sepharose beads not activated or conjugated to Poly I:C. These results demonstrated a consistency between the expression and activity of OAS1 enzyme (p46) in purified lysates from DU145 cell lines as compared to other prostate cell lines (Figs. 2 and 4).

### Wavelength Scans for Purified OAS Enzyme from DU145 Cell Lysate

**Fig. 5:**
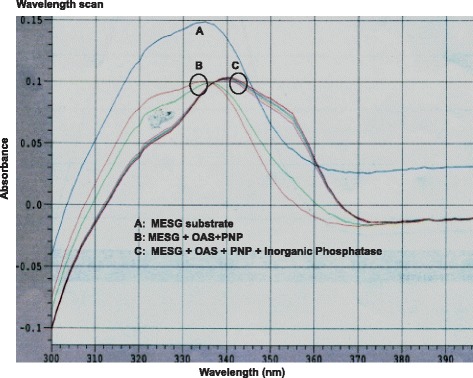
Wavelength Scan (300 nm–390 nm) for purified lysates from DU145 cell lines. A) MESG substrate only, B) MESG + OAS + PNP and C) MESG + OAS + PNP + inorganic phosphatase

As described in methods, the MESG substrate shows peak absorbance at 330 nm. As the reaction proceeds, the product formed has an absorbance shift that peaks at 360 nm. The data shown in Fig. 5 shows the expected wavelength shift from 330 nm to 360 nm for the enzymatic reaction on purified cell lysates from DU145 cells. These results indicated that the Poly I:C bound sepharose beads affinity purifies and activates OAS enzyme in DU145 cell lysates. The eluted purified lysate rich in OAS enzyme reacts with ATP in the elution buffer and polymerizes it to generate 2-5A and pyrophosphates. The pyrophosphate is further converted in to single phosphate molecules with the help of the enzyme pyrophosphatase. The phosphates react with the substrate MESG with peak absorbance at 330 nm and convert it to the product with peak absorbance at 360 nm, thus creating the shift in the absorbance peak and suggesting higher activity in purified DU145 cell lysates.

## Conclusion

This proposed method simplifies the detection/purification of OAS activity in prostate cancer cell line. The spectrophotometric method is a simple two step reaction based on measurement of pyrophosphates generated during ATP polymerization reaction in the presence of active OAS enzyme. The pyrophosphate is converted in to phosphate by inorganic phosphatase enzyme and the reaction of phosphates with its substrate MESG results in formation of purine based product causing shift in the absorbance from 330 nm to 360 nm. This technique has advantage over hazardous radio-labelling and time consuming methods with more than 4–5 step reactions to measure active OAS enzyme in prostate cells. The method can be applied to detect OAS activity from any cell/tissue with appropriate modification.

## Abbreviations

2-5 A: 2-5 oligoadenulate synthetase
AMP: adenine monophosphate
ATP: adenine triphosphate
BPE: bovine pituitary extract
DMEM: Dulbecco’s modified Eagle Medium
DNA: deoxy-ribonucleic acid
EGF: epidermal growth factor
GAPDH: glyceraldehyde 3-phosphate dehydrogenase
HEPES: (4-(2-hydroxyethyl)-1-piperazineethanesulfonic acid)
IFN: interferon
K-SFM: keratinocyte serum free medium
MESG: 2-amino-6mercapto-7-methylpurine ribonucleoside
NADPH: nicotinamide adenine dinucleotide phosphate
NaIO4: sodium periodate
OAS: 2-5 oligoadenylate synthetase
OAS1: oligoadenylate synthetase 1
PAGE: polyacrylamide gel electrophoresis
PAMPs: pathogen-associated molecular pattern
PEI: poly ethyleneimine
PNP: purine nucleoside phosphorylase
Poly I:C: polyinosinic:polycytidylic acid
PPi: pyrophosphate
RNA: ribonucleic acid
RNaseL: ribonucleic acid L
SARS: severe acute respiratory syndrome
SDS: sodium dodecyl sulfate
SNPs: single nucleotide polymorphisms
TLC: thin layer chromatography
